# The effects of practice distribution upon the regional oscillatory activity in visuomotor learning

**DOI:** 10.1186/1744-9081-6-8

**Published:** 2010-01-22

**Authors:** Bettina Studer, Susan Koeneke, Julia Blum, Lutz Jäncke

**Affiliations:** 1 Department of Neuropsychology, Psychological Institute, University of Zurich, Zurich, Switzerland; 2 Department of Experimental Psychology, University of Cambridge, Cambridge, UK; 3 Spinal Cord Injury Center, Balgrist University Hospital, Zurich, Switzerland

## Abstract

**Background:**

The aim of this study was to investigate the effects of a massed compared to a distributed practice upon visuomotor learning as well as upon the regional oscillatory activity in the sensorimotor cortex.

**Methods:**

A continuous visuomotor tracking task was used to assess visuomotor learning; the underlying neuronal correlates were measured by means of EEG. The massed practice group completed a continuous training of 60 minutes, while the distributed practice group completed four 15 minutes practice blocks separated by rest intervals.

**Results:**

While the massed and the distributed practice group did not differ in performance, effects of practice distribution were evident in the regional oscillatory activity. In the course of practice, the massed training group showed a higher task-related theta power and a strong task-related power decrease in the upper alpha frequency over the sensorimotor cortex compared to the distributed practice group.

**Conclusions:**

These differences in the regional oscillatory activity indicate a higher cognitive effort and higher attention demands in the massed practice group. The results of this study support the hypothesis, that a distributed practice is superior to a massed practice in visuomotor learning.

## Background

Motor skill learning is the process by which movements or sequences of movements come to be performed with strongly reduced effort through repeated intended practice [1]. Hence, practice plays a major role in the success of learning a new skill. Effects of varying different factors characterizing a practice schedule (e.g. absolute duration, intensity, distribution) have been investigated ever since the first studies in 1885 by Ebbinghaus in the field of learning and memory [2]. With respect to practice intensity or practice duration, previous studies indicate a clear positive relationship [3]. In contrast, previous findings are less clear regarding the distribution of practice. Ebbinghaus himself was the first to report distribution-of-practice effects by showing that better learning was achieved when the same amount of practice was distributed over two or more days compared to when the practice was completed in one single day [2]. More recent studies have approached this issue in the motor domain. In massed conditions, a motor task is practiced continuously, that is to say without any rest intervals; in distributed conditions the same amount of practice is divided into several blocks. A meta-analysis of 63 studies examining the effects of practice distribution in motor learning demonstrated the superiority of distributed practice and thereby confirmed Ebbinghaus's claim [4]. Interestingly, task complexity was found to serve as a moderating factor. For simple motor tasks the superiority of distributed practice was very strong (effect size d = 0.97), while a considerably smaller effect was observed for more complex motor tasks. The meta-analysis of Donovan and Radosevic [4] furthermore suggests that the length of the resting intervals in distributed practice designs plays an important role in determining the degree of its superiority over massed practice.

While meanwhile it is common knowledge that motor learning induces functional and anatomical changes within neural motor circuits [1,5], the interrelation between particular training parameters and the extent of training-induced neural changes is less clear. As mentioned above, the characteristics of the practice schedule (distributed versus massed practice) have been shown to play an important role in defining the outcome of motor learning. So far, explanations of the proposed superiority of distributed practice schedules were primarily based on practical considerations (e.g. attention demands, fatigue). The neural underpinnings of this effect, however, have not been investigated yet. Therefore, the principal aim of the present study was to investigate whether practice-dependent neural changes are influenced by temporal characteristics of the practice schedule. Conventional electroencephalography (EEG) was used to address this question in an experimental two-group design. One group practiced a bimanual visuomotor tracking task continuously for sixty minutes; while in the other group practice was distributed into four blocks of fifteen minutes with interspersed breaks.

Ever since the description of the alpha blockade by Hans Berger in 1924, it is known that neural activity influences the spectral composition of the EEG signal. In cortical motor areas, the power of frequency bands in the range of 10 to 20 Hz declines before and during the execution of movements as compared to a non-movement baseline condition - an effect referred to as task-related power decrease (TRPD) [6,7] or event-related desynchronization (ERD) [8]. This power suppression is assumed to reflect regional neural activity of motor cortical areas [9,10]. The extent of the power suppression has been observed to depend on several factors, such as the complexity of the movement, the required force and the movement rate. In the context of sensorimotor learning, TRPDs in the alpha and beta frequency ranges have repeatedly been reported to undergo changes across the period of practice [11-15]. While oscillations in the alpha frequency range are linked to somatosensory processing and integration, beta oscillations seem to be particularly sensitive to the motor components of a task [7,11,16-20]. Finally, there is some evidence, that task-related power in the theta frequency range can be affected by practice. In comparison to the alpha and beta frequencies, task-related theta power is less frequently investigated in motor learning research. Instead, task-related theta power was shown to be influenced by task difficulty, type of processing, memory load, cognitive effort, the ability to focus/sustain attention, alertness and practice [21-23]. Obviously many of these factors play a role in motor learning, too. Coombes and colleagues [21] have studied theta power in the motor learning context and observed a reduction of task-related theta power in the course of practicing a sequential motor task. The authors argue that this decrease in task-related power over the course of training reflects increasing task automaticity.

In the present study, participants were asked to perform a bimanual visuomotor task either following a massed or a distributed practice schedule. Based on the broad foundation provided by previous studies, we analysed task-related power changes to dissociate between the effects of the two practice schedules upon neural activity in the sensorimotor cortex. We hypothesise that over the course of practice both groups will show improvements in performance; but that the distributed practice group will perform better than the massed training group in later training stages. At the neural level, we assume that practicing the visuomotor task will lead to a diminished TRPD in the alpha and beta frequency bands, reflecting a reduction of motor-related activation due to increased task automaticity in both groups. We further hypothesise that TRPD changes in all three mentioned frequency bands observed across the practice period will differ between the two training groups, hence, reflecting the influence of practice distribution. Based on previous research, we particularly expect between-group differences in the theta frequency range. We hypothesise that the massed practice group will show a higher task-related theta power than the distributed practice group towards the end of the practice, reflecting a higher cognitive load and increased effort to maintain attention and perform accurately [4,21-27].

## Methods

### Participants

Thirty healthy right-handed female participants volunteered to participate in the present study (mean Age = 25.3 years, SD = 4.4 years). Handedness was assessed with the Annett-Handedness Questionnaire [28]. A standardized questionnaire was used to screen participants for neurological, psychiatric and medical exclusion criteria. The experiment took place at the Department of Neuropsychology, University Zurich, Switzerland. The study was approved by the local ethics committee and conducted in accordance with the Declaration of Helsinki. All participants gave written informed consent.

### Task

Participants were asked to practice a bimanual visuomotor tracking paradigm, which has been used in previous studies of our group [29]. In order to improve task-performance, participants had to learn to bimanually move a steering wheel according to a predefined movement pattern to minimize the difference between a changing foreground stimulus and a constant background stimulus. Participants were seated in front of a 17" monitor (resolution of 800 × 600 pixels), at a distance of approximately 1 m. The foreground stimulus consisted of a green-framed square of 50 pixels in the centre of the screen. The rest of the screen was coloured in grey and served as the background stimulus. Without any manipulation of the steering wheel, the target stimulus in the centre of the screen changed its grey tone according to a predefined pattern controlled by commercial experimental software (Presentation, Version 10.3, Neurobehavioral system, Albany CA, USA). The participants were not informed about the existence of this pattern, but simply told to keep the difference between the colour of the square and the background as small as possible. At the beginning of each trial, the foreground stimulus was presented in the same grey tone as the background stimulus. Then, the grey tone started to change its colour according to a sequence formed by 2000 data points. By continuously manipulating a commercial steering-wheel (SideWinder Force Feedback Wheel, Microsoft) the participants were able to influence the greyscale of the square and therefore counteract the change in colour. Hence, both the automatic colour change and the colour change induced by the subject simultaneously affected the foreground square in the middle of the screen. The brightness of the foreground square was parameterised according to a linear 256-step greyscale and refreshed 2000 times in the 33.33 second tracking time per trial. The refresh rate of the monitor was set to 60 Hz. The steering wheel registered movements between -125° and +125° with a precision of 9 bit (512 steps).

### Experimental design

The participants completed a total of 60 trials. For the statistical analysis, practice was split into groups of 15 trials corresponding to blocks 1 to 4. Each trial was initiated by the start signals "Achtung" (ready), "Fertig" (steady), "Los" (go) displayed in the middle of the screen. Subsequently the tracking commenced. Directly after each trial, participants were given feedback about their performance in form of a number displayed on the monitor indicating the mean deviation from the target track. Then a resting period of 16 seconds followed, during which a fixation cross was presented on screen. Thereafter, the next trial started automatically.

Participants were randomly assigned to one of two experimental groups in counterbalanced order. One group practiced according to a massed schedule. Here, the 60 trials were conducted at one stretch without rest. In contrast, the second group practiced according to a distributed schedule that allowed three breaks of 7.5 minutes between the four practice blocks (after the 15th, 30th and 45th trial). During the resting intervals, participants of the distributed training group were presented with a commercial radio play via standard headphones (Technics Stereo Headphones RP-F550). Participants were instructed to pay attention to the radio play and press a button each time a particular character was speaking. This task was applied to prevent intentional rehearsal of the tracking movements during the rest intervals.

### Data acquisition

Continuous EEG was recorded from 30 surface silver-silver chloride electrodes, positioned in accordance with the international 10-20 system and mounted with the "Easy Cap System" (FMS Falk Minow Services, Herrsching-Breitbrunn, Germany). The recording was referenced to FCz and impedances were kept below 5 kΩ. The BrainVision amplifier system and the BrainVision Recorder software (BrainProducts, Germany) were used to record the data. The electrooculogram was registered by two additional electrodes located below the outer canthi of each eye. The signal was sampled at 500 Hz and bandpass-filtered from 0.5 - 70 Hz. Prior to the first and subsequent to the last practice block, five minutes of spontaneous EEG with the alternating conditions "eyes open" and "eyes closed" were recorded.

### Data analysis - behavioural data

The steering-wheel position at each of the 2000 data points forming the sequence was compared with the required target position. The mean absolute deviation per trial was then calculated by averaging over the registered deviation values from the 2000 data points of each trial using Matlab 6.5 (The MathWorks, Inc., USA). The further analysis of the behavioural data was performed using statistical analysis software SPSS (Version 13.0, SPSS Inc., USA). Outliers were defined as trials in which the performance differed more than two standard deviations from the mean performance of the practice block and excluded from further analysis. We then recalculated the mean performance per practice block by averaging over the remaining trials of each block. Participants that showed a significant higher performance in block 4 compared to block 1 were classified as learners (one-tailed independent samples t-test p < 0.05), while participants that did not improve significantly were classified as non-learners and excluded from further analysis [30].

Finally, a repeated-measures ANOVA with 'block' (practice blocks 1 - 4) as within-subject factor and 'group' (massed training vs. distributed training) as between-subject factor was conducted. Greenhouse-Geisser corrections were used to prevent effects of heteroscedasticity. To further investigate the emerging main effects, subsequent t-tests were performed.

Since *P*-values strongly depend on sample size we furthermore calculated effect size measures to obtain information on how strong an effect is. ETA^2 ^(η^2^) is reported in multivariate ANOVA statistics and describes the variance attributed to the independent variable of interest. For the t-tests, Cohen's *d *[31] was determined (*d *= M1 - M2/*σ *_pooled_), that is, the difference between two means divided by the pooled standard deviation. The pooled standard deviation is the square root of the average of the sample variances [32]. According to Cohen [31] an effect size of *d *> 0.2 is considered as being small, an effect size of *d *> 0.5 is considered as being moderate and an effect size *d *> 0.8 is considered as being large.

### Data analysis - electrophysiological data

The EEG raw data were analysed offline using the BrainVision Analyzer software package (Brain Products GmbH, Germany). First, a bandpass filter from 1.5 to 30 Hz was applied, then the data were resampled and re-referenced to an average reference. In order to avoid considerable loss of data due to the rejection of artefact-contaminated EEG epochs, we ran an independent component analysis (ICA) to correct for eye artefacts. The data were carefully checked for additional artefacts by visual inspection. The continuous EEG was then segmented into four blocks and further subdivided into the 15 trials per block. Each trial was split into a movement segment of 33 seconds and a resting segment of 14 seconds. The movement segments were defined as the "activation condition". The resting segments relate to the time periods when the fixation cross was presented and no movement occurred.

As this study focused on learning-related changes in the activity of the sensorimotor cortex, two clusters of electrodes of interest (EOI) were defined in accordance with previous EEG studies on motor learning and neuroplasticity [7,15,33]. We selected electrodes that overlie the sensorimotor cortex of the left (FC3, C3, CP3) and right (FC4, C4, CP4) hemisphere. Only data recorded from these electrodes of interest were considered for the statistical analysis.

For the analysis of task-related power, the movement and resting segments were further segmented into epochs of 1000 data points (corresponding to 2 seconds); artefacts-contaminated epochs were excluded. For spectral power analysis a Fast Fourier Transformation (FFT) including the application of a Hanning window was computed for each of the 2s epochs and all electrodes. The power spectrum from 1 - 30 Hz was calculated for each single epoch and then averaged across all epochs of each block. Next, the averaged power spectra obtained from the six selected electrodes were pooled according to the above-mentioned EOI cluster definitions. Then, a mean power value was extracted for each frequency band (theta, lower alpha, upper alpha, lower beta, upper beta), EOI (left, right), experimental block (block 1 - 4) and condition (movement, resting). Logarithmic task-related power (logTRP) was then calculated for each frequency band, EOI and experimental block according to the following formula: logTRP = log (Power movement) - log (Power rest). Task-related power decreases are therefore expressed as negative values while task-related augmentations in power are expressed as positive values. The logarithmic transformation was applied in order to stabilize the variance of spectral power estimates [34].

In accordance with previous research of our group [29,30], task-related power was calculated for five individually defined frequency bands. The four individual frequency bands covering the alpha and beta ranges were defined using the following procedure: At first, the individual alpha peak was determined for each subject: The power spectra pooled over the two lateral EOIs were compared between the movement and the resting periods (averaged across the whole experiment) and the individual alpha peak (iAP) was defined as the frequency showing the strongest difference. Then, four frequency bands were defined using the iAP as an anchor: 1) lower alpha band α1 = iAP to iAP-4 Hz, 2) upper alpha band α2 = iAP to iAP+2 Hz, 3) lower beta band β1 = iAP+2 Hz to iAP+10 Hz and 4) upper beta band β2 = iAP + 10 Hz to iAP+18 Hz. Finally, we used the individually detected transition frequency to define the individual theta band, a method introduced by Klimesch [24]. The transition frequency (TF) marks the change from theta activity to alpha activity. To identify the TF the power spectra pooled over all 32 electrodes were compared for the moving and the resting period. The TF can then be determined by identifying the frequency where the two power spectra intersect. The fifth frequency band was defined: 5) theta band θ = TF - 2 Hz to TF.

For the two EOI clusters and the different frequency bands, the data were analyzed in separate repeated-measure ANOVAs with 'block' (practice blocks 1-4) as within-subject factor and 'group' (massed training vs. distributed training) as between-subject factor. Greenhouse-Geisser adjustments were conducted to protect from effects of heteroscedasticity. To further investigate the emerging main effects, subsequent t-tests were performed.

Since *P*-values strongly depend on sample size we furthermore calculated effect size measures to obtain information on how strong an effect is. ETA^2 ^(η^2^) is reported in multivariate ANOVA statistics, while Cohen's *d *[31] is reported for the t-tests.

## Results

30 participants participated in this experiment. Six participants had to be excluded from the statistical analyses; four of them due to technical interferences during the data acquisition. Two participants showed extreme values in the overall tracking performance, which led us to assume that they failed to understand the task. Two participants could not be regarded in the group analysis because no clear individual alpha peak could be identified. From the remaining 22 participants, six were classified as non-learners and therefore also excluded from the group analysis. In the end, the massed practice group consisted of 9 participants while the distributed practice group contained 7 participants.

### Behavioural data

**Figure 1:**
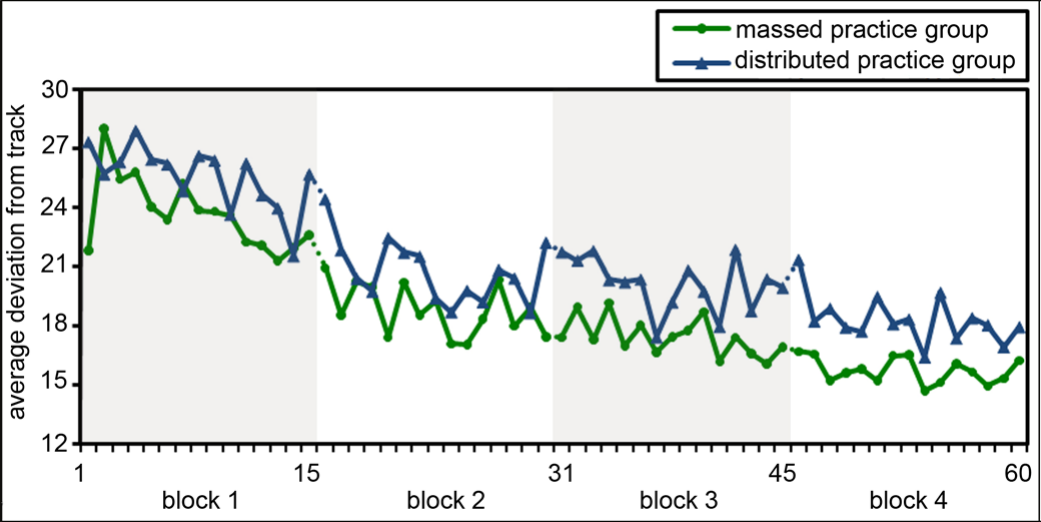
**Motor performance**. Average performance per trial in the two experimental groups; lower values reflect a higher performance.

The repeated measures ANOVA revealed a clear main effect of 'block' (F = 43.19, p < 0.001, ηp^2 ^= 0.76). Post-hoc t-tests showed significant increases in performance between each block and its subsequent block (pairwise comparisons, one-tailed t-tests, p < 0.05). No significant effect for the between-subject factor 'group' (F = 1.24, p = 0.28, ηp^2 ^= 0.08) nor a significant 'group × block'-interaction (F = 0.17, p = 0.84, ηp^2 ^= 0.01) were observed, indicating that both experimental groups performed equally well throughout practice. The detailed learning curves for the massed and the distributed practice group are presented in Figure 1.

### Electrophysiological data

The execution of tracking movements was accompanied by power changes over the sensorimotor cortex in the alpha, beta and theta frequencies compared to rest. In all practice blocks and both clusters of EOIs, a task-related power decrease (TRPD) was found in the upper alpha (α2) and lower beta (β1) frequencies (paired two-tailed t-tests, p < 0.05, d = between -0.53 and -1.09, please refer to Table 1 for details regarding the statistical comparisons). As expected, α2 and β1 power were lower during the movement phase compared to the resting phase. Also according to our hypotheses, a task-related power increase (TRPI) was observed in the theta frequency band (θ) in all practice blocks and both clusters of EOI (paired two-tailed t-tests, p < 0.001, d > 0.80, see Table 1). In other words, theta power was higher during the movement period compared to the resting period.

**Table 1:** Task-related power changes

Left EOI				
	**Block 1**	**Block 2**	**Block 3**	**Block 4**

α1	**T(15) = 2.6, p = .02, d = .34**	T(15) = 1.8, p = .09, d = .27	**T(15) = 2.3, p = .04, d = .25**	T(15) = 2.1, p = .06, d = .28

α2	**T(15) = -5.8, p = .00, d = -1.09**	**T(15) = -5.2, p = .00, d = -.98**	**T(15) = -4.6, p = .00, d = -.85**	**T(15) = -4.3, p = .00, d = -.87**

β1	**T(15) = -5.0, p = .00, d = -.89**	**T(15) = -3.5, p = .00, d = -.68**	**T(15) = -3.6, p = .00, d = -.61**	**T(15) = -2.9, p = .01, d = -.54**

β2	T(15) = 0.9, p = .36, d = .11	T(15) = 1.2, p = .27, d = .12	T(15) = 1.1, p = .29, d = .10	T(15) = 0.4, p = .70, d = .04

θ	**T(15) = 4.2, p = .00, d = .85**	**T(15) = 4.4, p = .00, d = .88**	**T(15) = 4.1, p = .00, d = .80**	**T(15) = 4.2, p = .00, d = .86**

**Right EOI**				

	Block 1	Block 2	Block 3	Block 4

α1	**T(15) = 2.3, p = .04, d = .27**	**T(15) = 2.3, p = .03, d = .27**	T(15) = 2.1, p = .06, d = .21	**T(15) = 2.2, p = .04, d = .26**

α2	**T(15) = -4.9, p = .00, d = -.93**	**T(15) = -4.7, p = .00, d = -.90**	**T(15) = -4.6, p = .00, d = -.78**	**T(15) = -3.9, p = .00, d = -.81**

β1	**T(15) = -4.2, p = .00, d = -.82**	**T(15) = -3.9, p = .00, d = -.79**	**T(15) = -3.2, p = .00, d = -.61**	**T(15) = -2.7, p = .01, d = -.53**

β2	T(15) = 0.7, p = .50, d = .07	T(15) = 0.7, p = .50, d = .07	T(15) = 0.7, p = .51, d = .05	T(15) = 0.0, p = 1.00, d = .00

θ	**T(15) = 5.5, p = .00, d = .85**	**T(15) = 7.9, p = .00, d = .91**	**T(15) = 6.1, p = .00, d = .89**	**T(15) = 5.1, p = .00, d = .85**

Statistical comparisons (paired two-tailed t-tests) between resting and activation periods for the 5 frequency bands, 2 EOIs and 4 practice blocks. Significant differences (TRPDs in case of α2 and β1/TRPIs in case of α1 and θ) are indicated in bold type.

Unexpectedly, a task-related power increase was also observed in the lower alpha (α1) frequency bands in most blocks and both EOIs (paired two-tailed t-tests, p < 0.05). That is to say, the lower alpha power was higher during rest than during movement throughout the training. In the upper beta (β2) frequency, the power did not differ between the movement period and the resting period in any of the blocks (paired two-tailed t-tests, p > 0.05). This indicates that the execution of the task had no effects on β2-power. The power in β2 was therefore not analysed regarding training and group effects.

For each of the other four frequency bands, repeated measures ANOVAs with the within-subject factor 'block' and the between-subject factor 'group' were conducted separately for each EOI cluster.

#### General training effects (over all participants)

**Figure 2:**
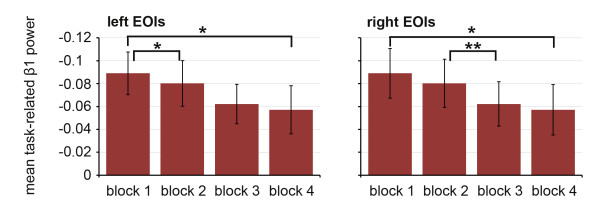
**Group-independent effect of practice**. Mean task-related power in lower beta band measured at electrodes overlying the left and the right sensorimotor cortex, * = p < 0.05, ** = p < 0.01. Error information is given as SE.

Across all participants, a significant main effect of 'block' was found in the β1 frequency band in the right EOI (F = 4.285, p < 0.05, ηp^2^= 0.24) and the left EOI (F = 3.71, p < 0.05, ηp^2^= 0.21). Post-hoc comparisons between the first and the last practice block showed a significant attenuation of the β1-TRPD over the course of practice in both EOI clusters (paired one-tailed t-tests, p < 0.05, d = -0.44 (right EOI), d = -0.37 (left EOI)). As illustrated in Figure 2, the main effect of 'block' primarily results from differences between the first two practice blocks in the left EOI (left EOI: T(15) = -2.7, p = 0.01, d = -0.31 ; right EOI: T(15) = -1.0, p = 0.16, d = -0.10) and between the second and the third block in the right EOI (right EOI: T(15) = -3.3, p = 0.00, d = -0.22, left EOI: T(15) = -0.9, p = 0.19, d = -0.08), while differences between blocks 3 and 4 were less prominent (right EOI: T(15) = -0.5, p = 0.31, d = -0.06, left EOI: T(15) = -0.5, p = 0.33, d= -0.06).

No significant main effects or trends of 'block' were found in the other four analysed frequency bands.

#### Between-group differences

The repeated measures ANOVAs revealed a significant effect of 'group' in the α2 frequency band in the right EOI (F = 4.70, p < 0.05, ηp^2 ^= 0.25). A similar trend was observed in the left EOI cluster, however the effect marginally failed to reach significance (F = 3.56, p = 0.08, ηp^2 ^= 0.20). Post-hoc analyses revealed that while both practice groups showed a TRPD in α2 in all blocks, this TRPD was stronger in the massed practice group compared to the distributed practice group in both clusters of EOI. When conducting post-hoc t-tests, the difference becomes significant in block 2 (left EOI: T(14) = -2.2, p = 0.04, d = -1.12; right EOI: T(14) = -2.6, p = 0.02, d = -1.33). We furthermore find a trend for block 4 (right EOI: T(14) = -2.0, p = 0.07, d = -1.00).

**Figure 3:**
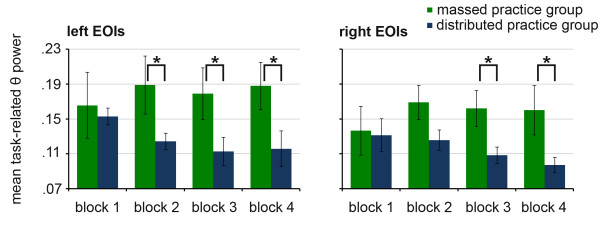
**Effect of practice distribution**. Mean task-related theta power over the left and the right sensorimotor cortex, * = p < 0.05. Error information is given as SE.

Although the between-group differences in the theta frequency band failed to reach significance in both clusters of EOI (left EOIs: p = 0.14, right EOIs: p = 0.11), visual inspection of the data and the results from the ANOVAs pointed towards different developments of task-related theta power over the course of practice in the two practice groups. Therefore post-hoc analysis was nevertheless conducted. Independent t-tests revealed significantly higher TRPIs in the theta band for the massed compared to the distributed practice group in blocks 2 (T(9.3) = 1.9, p < 0.05, d = 0.89), 3 (T(14) = 1.8, p < 0.05, d = 0.90) and 4 (T(14) = 2.0, p = 0.03, d = 1.01) in the left EOI and in block 3 (T(11) = 2.4, p = 0.02, d = 1.14) and 4 (T(9.5) = 2.1, p = 0.03, d = 1.01) in the right EOI. As displayed in Figure 3, these results indicate a different development of task-related θ power over the course of practice in the two experimental groups: While both practice groups show an increase in theta power during the movement period compared to the rest period, this TRPI grows stronger over the course of practice in the massed practice group, while it declines over the course of practice in the distributed practice group.

No significant differences between the two groups regarding the task-related power over the course of the experiment were found in the lower alpha or the lower beta frequency bands.

## Discussion

The primary aim of this study was to investigate the effects of practice distribution upon the behavioural improvements in a visuomotor tracking task as well as upon the underlying neuronal activation patterns. We found that the general improvement in task performance over the course of practice was accompanied by a gradual attenuation of the task-related power decrease in the lower beta band at electrodes overlying lateral motor areas over the right and the left lateral sensorimotor cortex. Second, although the massed and distributed practice group did not differ with respect to performance and learning profile, the patterns of regional oscillatory activity in the upper alpha and theta frequency bands developed differently in the two groups over the course of practice.

### Behavioural data

The analysis of the behavioural data from all participants revealed a clear improvement in performance over the course of practice. In line with the previous literature about the temporal properties of motor learning [1,35] a quick improvement was observed in the early stages of the practice, while the learning curve became flatter in later learning stages. Given that the tracking error values decreased until the last trial, we assume that learning continued until the end of practice. Six participants did not show a significant improvement in their performance over the course of the practice. This finding is consistent with a previous study using a similar visuomotor tracking paradigm [30]. Blum and colleagues also report a relatively high percentage of non-learners. The reasons why some participants failed to improve in the course of practicing this particular task are not entirely clear. Various explanations, such as failure to understand the task instruction, lack of motivation, task difficulty but also an interfering attempt to learn the tracking movements explicitly, are conceivable.

On the basis of previous research about the distribution-of-practice effect [see 4 for a review], we hypothesized that participants following a distributed practice would show a stronger improvement in performance in later practice sessions as compared to the massed practice group. The behavioural data of the present study, however, do not support this hypothesis. The massed and the distributed practice group performed equally well and showed an equal amount of improvement over the course of practice. The complexity of the task used in this study may provide an explanation for this inconsistency with the findings of most previous studies. The superiority of distributed over massed practice has predominately been shown in simple motor tasks in which the temporal distribution of training sessions led to better task performance and/or longer retention [4,36-38]. A meta-analysis by Donovan and Radosevich [4] showed that overall task complexity is significantly correlated with the effect size of practice distribution on performance. When only studies using difficult motor tasks where analysed, only a weak distribution of practice effect was found. Several factors, such as task characteristics, the finding of continuous improvement in performance until the end of the rather long practice session and the presence of non-learners, indicate that the bimanual visuomotor tracking paradigm used in this study is a rather difficult motor task.

It can also be argued that the distributed practice group did not show a superior performance compared to the massed one due to the design of the practice schedules. The length of the rest interval between practice blocks in the previous literature varies between minutes and days and an ideal rest interval has not yet been found [38]. The meta-analysis by Donovan and Radosevich [4] furthermore shows that the optimal length of the breaks in distributed practice schedules is highly dependent on the particular task performed and that there might be an interaction between the optimal length of the rest interval and task complexity. It can be speculated that, for the task used in the present study, the rest intervals were too short to be of benefit. The question whether different variations of the amount and length of pauses and practice blocks might lead to distribution-of-practice-effects remains to be addressed in future experiments.

Another possible explanation for the contrast between the finding of this study and some previous studies consists in the time point of measurement. Some of the previous studies have assessed the final skill level in a delayed retention test rather than at the end of the practice session. Dail and Christina [36] analysed the performance of a massed and a distributed practice group in a golf putting task during practice and in retention test. The authors observed a higher performance in the distributed training group in the last practice session, but an even stronger superiority of the distributed group in the retention tests. Hence, practice distribution may have a higher impact on the retention performance than on the acquisition performance in motor learning. In the present study retention was not assessed. It would be of interest to clarify this question in a future experiment that includes a retention test with some delay after the completion of the practice.

Finally, it is conceivable that the practice of the distributed group would have to be spread over several days in order to be more effective than massed practice. Although Mackay et al. [37] found that a distributed practice within one day can lead to a higher performance in the acquisition of a fine motor skill, most other studies investigating the distribution of practice effect were using a design in which training was distributed over several days [36,39]. Shea and colleagues [38] compared two different designs of distributed practice schedules in two experiments about the learning of continuous and discrete motor tasks. Their results support the hypothesis that a distributed practice may be more efficient when spread over several days instead of completed within one day.

### Electrophysiological data

In accordance with previous ERD studies of motor behaviour [14,34,40], the execution of tracking movements was accompanied by task-related power changes in lower and upper alpha, lower beta and theta over the sensorimotor cortex. As expected, some of these task-related changes in the regional oscillatory activity were systematically modified over the course of practice. In line with our hypothesis, the improvements in performance were accompanied by a reduction of the TRPD in the lower beta band over the course of practice. Our results furthermore showed that like the behavioural changes, the strongest changes in task-related beta power took place in the early phases of practice. This finding is consistent with the result of a recent study by Kranczioch and colleagues [14], where an attenuation of the TRPD in fronto-central beta was found following practice of a visually guided power-grip task. We argue that this reduction of TRPD in the lower beta band reflects a gradual reduction of motor-related cortical activation. In other words, the observed attenuation of TRPD likely represents a correlate of the effect that task execution becomes less effortful and less attention demanding as a result of increasing task automaticity. This interpretation has previously been supported by other authors investigating the neuronal correlates of motor learning [14,41,42].

Contrary to our expectations, a slight TRPI was found in the lower alpha band. The lower alpha band is thought to reflect general task demands and attention processes [23,34]. This finding is somewhat surprising as typically a TRPD is expected during the execution of a task compared to rest. Further research is needed in order to determine what might have caused this slight TRPI in lower alpha.

In the theta band, no systematic changes were observed over the course of practice when all learners regardless of the practice group were analyzed. Interestingly, however, we found a number of differences between the two groups with respect to practice-induced changes of the regional oscillatory theta activity. While the TRPI in theta decreased over the course of practice in the distributed practice group, the TRPI raised over the course in the massed practice group. In other words, the massed practice group showed a higher TRPI in theta than the distributed practice group in later training block, in accordance with our hypothesis. The finding of a higher task-related theta power in later training sessions in the massed practice group is in line with the previous literature linking increased theta power with increased cognition demand, increased attentional demands and increased effort [21-27,21-24,26]. We argue that a massed practice leads to a higher cognitive load and an increased effort to maintain and focus attention compare to a distributed practice. In the distributed practice, the rest intervals may allow the participants to recover.

Furthermore, the distributed group displayed a weaker task-related power decrease in upper alpha in both clusters of electrodes in the second practice block. While lower alpha is thought to reflect general cognitive demands and attention processes, previous studies indicate that upper alpha desynchronization is related to task-specific aspects, such as for example certain aspects of motor processing [43]. Manganotti and colleagues [40] analysed task-related power changes during the execution of finger movement sequences of increasing complexity and found that the magnitude and spatial extent of the task-related power decreases in the upper alpha band over the sensorimotor cortex were stronger for sequences with higher complexity compared to simple sequences. Klimesch and colleagues [23,24] furthermore observed that task-related upper alpha power differed between good and bad performers in a semantic memory task. While the task complexity was initially the same for both practice groups, it is conceivable that the weaker task-related power decrease in upper alpha observed in the distributed practice group in the second block, that is to say after having taken a break, reflects reduced cognitive demands and increased task-automaticity.

### Limitations

Some limitations of the present study should be noted. Firstly, all participants were female and right-handed. Future research should replicate the main findings in independent samples. Secondly, our study was focused specifically on the effects of different practice distributions upon the local activity of the sensorimotor cortex. It is conceivable that a prolonged training of a visuomotor task might additionally lead to functional changes in brain areas outside the sensorimotor cortex. This hypothesis should be tested in forthcoming studies.

## Conclusions

This study examined the effects of a massed compared to a distributed training in visuomotor learning. In the behavioural data no differences between the two groups were evident and therefore the superiority of a distributed practice could not be confirmed by the behavioural data. However, the results of this study confirm distribution of practice-effects in motor learning on the neurophysiologic level. The analysis of regional oscillatory activity indicates a higher cognitive load and increased attentional demands in the massed training group compared to the distributed training group towards the end of the practice session. It is conceivable that an elongation of the practice session would lead to exhaustion in the massed practice group resulting in a weaker learning compared to the distributed practice group. Therefore, the results of this study generally support the hypothesis, that a distributed practice is superior to a massed practice when trying to acquire a motor skill.

## Competing interests

The authors declare that they have no competing interests.

## Authors' contributions

BS contributed to conception and design of the study, took care of the data acquisition, performed the data analyses and interpretation and drafted the manuscript. SK contributed to the design of the study, participated in data interpretation and helped drafting the manuscript. BJ participated in the design and helped with data processing and statistical analyses. LJ contributed to the design of the study and critically revised the results. All authors read and approved the final manuscript.
